# Computed tomography-guided needle biopsy - the crucial diagnosis of head and neck masses

**DOI:** 10.1590/0100-3984.2021.54.6e2

**Published:** 2021

**Authors:** Ana Célia Baptista Koifman

**Affiliations:** 1 Universidade Federal do Estado do Rio de Janeiro (Unirio)Rio de Janeiro, RJ, and Universidade Federal do Rio de Janeiro (UFRJ), Rio de Janeiro, RJ, Brazil.

Imagine a patient with head and neck cancer (HNC). The tumor is located in one of the multiple complex anatomical spaces of the region, with imminent locoregional invasion of vital structures. The mutilation can come from the disease itself or from the treatment, which is often heroic and even, why not say, merciful. The debilitation is often great, and the expectations can be inglorious. An accurate and rapid diagnosis is imperative, to attempt a salvage therapy. In general, the aim is to minimize the devastating aesthetic and functional consequences, as well as to reduce the suffering of the patients and their families. In view of this worrisome scenario, Amoedo et al.^(^1^)^, in an article published in the previous issue of Radiologia Brasileira, presented a retrospective analysis of 68 patients with HNC who underwent computed tomography-guided percutaneous needle biopsy (core biopsy). Most of the lesions were located in the suprahyoid region, and most of the tumors were found to be squamous cell carcinomas. The procedure was shown to have a specificity of 100% and a diagnostic accuracy of 96.6%. Only a few, self-limited, complications were reported, which is in keeping with the literature. The authors concluded that the procedure is safe and effective for defining the histological diagnosis.

Squamous cell carcinoma accounts for 90% of malignant tumors in the head and neck region^(^2^)^, with an estimated five-year survival rate of 40-50%. More than two thirds of patients diagnosed with squamous cell carcinoma have locally advanced disease at the time of diagnosis. Recurrence rates are reported to be as high as 60% within the first two years after treatment, and 20-30% of patients develop distant metastases^(^3^)^. It is noteworthy that histopathologically benign lesions can be as challenging as are their malignant counterparts, either because of the space they occupy and the feasibility of their resection or because of the possibility of postoperative sequelae.

Computed tomography-guided biopsies are generally reserved for lesions found at a depth greater than 3 cm from the skin surface, near the skull base, in the parapharyngeal or retropharyngeal spaces, in the deep lobe of the parotid gland, or in the pterygopalatine fossa^(^4^)^. There are two other types of procedures for obtaining material for histological analysis, each with its own advantages and limitations—fine needle aspiration and surgical biopsy—and other imaging methods used in guiding those procedures, as ultrasound and positron emission tomography/computed tomography^(^1^)^. Unfortunately, no procedure is 100% safe and effective. Biopsies can fail for many reasons, such as insufficient material, an inconclusive diagnosis, and histological findings that are discordant with the morphological pattern of the lesion seen on imaging. All of these factors can delay treatment and have a negative impact on survival. It is no less important to avoid performing multiple procedures, which generate stress and anxiety for the patients, as well as increasing the possibility of complications. There is also a non-negligible risk of tumor cell seeding along the needle tract^(^5^)^.

Few existing studies can explain the lack of definitive, congruent scientific evidence to guide the management of cases of HNC. Studies of the topic have comprised small, heterogeneous patient samples, have varied greatly in terms of the reported size and location of the lesions, and have encompassed a vast group of histological diagnoses, as well as including reports of untreated neoplasms, residual disease, and recurrence. The diverse therapeutic arsenal, which includes isolated and combined therapies, creates an additional dilemma and makes comparisons across studies difficult, as well as thwarting attempts at standardization.

In the context of so many knowledge gaps and to overcome limitations, human and technological contributions must remain interconnected and must be based on excellence in multidisciplinary interaction and multiparametric imaging, in order to allow better optimization and individualization of the diagnostic-therapeutic process.

The consequences for patient quality of life and the prognosis of the disease after core-biopsy remain poorly defined. Therefore, there is an urgent need for further prospective and follow-up studies with multicenter collaborations and analyses of the reproducibility of the methods. Other more recent approaches, such as liquid biopsy^(^6^)^, which is a rapid and noninvasive procedure (saliva being an additional resource in HNC), and artificial intelligence algorithms^(^7^)^, which aggregate radiological, histological, and molecular data, should provide promising and more complete information and will bring the future ever closer.
